# Exploring Sustainable Diet Drivers: An Extended TPB Approach to Alternative Protein Acceptance in Southern Italy

**DOI:** 10.3390/nu17243942

**Published:** 2025-12-17

**Authors:** Gennaro Civero, Gennaro Punzo, Debora Scarpato

**Affiliations:** Department of Economic and Legal Studies, University of Naples Parthenope, via Generale Parisi 13, 80132 Naples, Italy; gennaro.civero@collaboratore.uniparthenope.it (G.C.); gennaro.punzo@uniparthenope.it (G.P.)

**Keywords:** sustainable food choice, consumer behaviour, moral and social norms, structural equation modelling, meat substitutes, insects, cultured meat, plant-based proteins

## Abstract

**Background/Objectives**: This study investigates how consumers decide to adopt alternative proteins—specifically insect-based, cultured meat, and plant-based options—as part of a transition towards environmentally sustainable diets. Building on an extended Theory of Planned Behaviour (TPB), the analysis adds personal moral norms and environmental concerns to better capture the ethical and normative drivers of food choice. **Methods**: Survey data from 948 residents of the Campania region (southern Italy) were analysed using partial least squares structural equation modelling (PLS-SEM) to assess the relationship among classical TPB constructs, personal moral norms, environmental concerns, and behavioural intention towards alternative protein consumption. **Results**: Personal moral norms emerge as the strongest predictor of behavioural intention, directly and indirectly influencing attitudes and environmental concerns. Subjective norms also affect intention, primarily by reinforcing moral norms and perceived behavioural control, although their direct impact is not significant. Classical TPB constructs show limited direct effects. **Conclusions**: The findings suggest that consumers’ sustainable food intentions are more strongly shaped by moral identity and the surrounding social context than by attitudes alone. The evidence supports the development of culturally sensitive strategies designed to strengthen moral and normative motivations and foster the adoption of alternative proteins.

## 1. Introduction

The global population is projected to reach 9.1 billion by 2050, with most of this growth occurring in developing countries [1]. This demographic expansion will require a 70% increase in global food production, corresponding to an additional 1 billion tons of cereals and 200 million tons of meat. Rising population and disposable incomes have already driven a substantial increase in global meat consumption, intensifying environmental challenges such as climate change, greenhouse gas emissions and the overall carbon footprint [1,2,3,4]. Alongside environmental concerns, public health and ethical considerations have intensified societal and policy pressure to reduce meat consumption [5,6,7].

For this reason, international institutions, such as the United Nations through the Sustainable Development Goals and the Food and Agriculture Organization (FAO), have called for reduced red meat intake and the adoption of more sustainable diets [8,9]. 

Meeting the nutritional needs of a growing global population in a sustainable manner has therefore increased interest in alternative protein sources. In Europe, consumer demand for environmentally friendly foods—particularly meat alternatives—has grown steadily [10]. Within this context, alternative proteins have emerged as promising options to conventional meat, typically classified in the literature into three main typologies:

(i) Insects, which are rich in protein, fibres, unsaturated fats and essential micronutrients such as iron, zinc, and B vitamins [11]. Furthermore, insect farming requires fewer resources such as land and water and has a lower global warming potential, despite its energy demands [11]. Nevertheless, consumers in industrialised countries, especially Europe and North America, are reluctant to incorporate insects into their diets [11,12,13,14,15].
(ii) Cultured meat, which offers environmental and ethical benefits. It is produced using techniques such as stem cell isolation, cell culture and tissue engineering which allow for the growth of muscle fibres outside of animals [16]. Many studies have focused on the characteristics of cultured meat [17,18,19], while others highlight that many consumers are concerned about its price, sensory quality and safety of cultured meat [19,20]. More recently, some studies have compared cultured meat with other protein sources [21].
(iii) Plant-based proteins, which have progressively gained acceptance as sustainable meat substitutes, in part due to greater public awareness of environmental sustainability [22,23,24,25]. Furthermore, plant-based meat alternatives are considered nutritionally adequate [26].

The success of these products in reducing meat consumption depends heavily on consumer acceptance [21]. This is particularly true for entomophagy—the consumption of insects—which in certain cultures is a traditional and nutritious dietary component [27]. Despite the growing interest in alternative proteins, the current literature shows several limitations. First, most studies emphasise product characteristics, their nutritional value and their environmental impact, while psychological aspects have received comparatively less attention. Secondly, the existing literature tends to examine a single category of alternative proteins at a time and only a small number of studies consider all three types within a single analysis. Thirdly, empirical evidence from southern European contexts is still limited, despite the fact that local gastronomic traditions can significantly influence consumers’ openness to innovative foods.

Behavioural theory models offer useful tools for analysing the adoption of sustainable food practices. Specifically, this study aims to identify the factors that drive or inhibit consumers’ behavioural intentions to adopt alternative proteins. To this end, it adopts an extended version of the Theory of Planned Behaviour (TPB) [28] that incorporates the traditional constructs—attitudes, subjective norms, and perceived behavioural control—together with two additional constructs, namely, personal moral norms and environmental concerns, to fully capture the moral and ecological dimensions of sustainable food choices. This integrated framework is empirically tested using partial least squares structural equation modelling (PLS-SEM), which assesses both direct and indirect pathways through which these factors influence consumers’ intentions to adopt alternative proteins.

In light of the identified theoretical and empirical gaps, this study pursues the following objectives:

1. Examine the psychological and social determinants (attitudes, subjective norms, perceived behavioural control, moral norms and environmental concerns) that influence consumers’ intention to adopt alternative proteins—edible insects, cultured meat, and plant-based proteins—using an extension of the Theory of Planned Behaviour.
2. Explore the role of the Mediterranean cultural context with reference to the Campania region, on consumers’ openness to innovative and sustainable foods.

The Campania region was deliberately selected as the case study due to its distinctive cultural, historical, and gastronomic identity, which makes it particularly relevant for research focused on sustainable food systems and dietary behaviour. First, the region includes Cilento, a UNESCO-recognised area that played a fundamental role in the development and promotion of the Mediterranean diet, widely considered a cultural heritage and a model of sustainable nutrition. Second, with a total of fifty Michelin-starred establishments in the 2025 Michelin Guide, the region expresses a unique balance between local roots and culinary innovation, placing it at the forefront of the national and international restaurant scene. The presence of one three-star restaurant, along with seven two-star restaurants and the widespread distribution of one-star restaurants throughout the region, is not only an indicator of gastronomic excellence but also reflects the vitality and complexity of Campania’s culinary heritage. From this perspective, the region certainly embodies what the literature defines as gastronomic heritage [29,30], a set of knowledge, symbols and practices in which food becomes a vehicle of identity, memory, and cultural continuity. At the same time, Michelin starred restaurants play a significant educational role, representing privileged places of innovation and the transmission of professional skills. Third, the high quality of regional cuisine is closely linked to the availability of high-quality raw materials. Campania is, in fact, one of the Italian regions with the richest and most diverse certified agri-food heritage with numerous iconic local products recognised under the European Union’s official quality schemes such as Protected Designation of Origin (PDO), Protected Geographical Indication (PGI), and Traditional Speciality Guaranteed (TSG). Fourth, the combination of certified products, layered culinary traditions and starred restaurants contribute to creating a distinctive foodscape capable of also acting as a driver of tourist attraction.

The remainder of this paper is structured as follows: Section 2 outlines the theoretical framework and research hypotheses. Section 3 describes the methodological approach and data collection process. Section 4 presents the results, and Section 5 reports the discussion of the findings and outlines the study’s limitations. Section 6 presents the conclusions, the policy implications and directions for future research.

## 2. Theoretical Framework and Research Hypotheses

Psychological theories offer two primary perspectives on why individuals engage in environmentally responsible behaviour [31]. The first perspective, represented by frameworks such as the Norm Activation Theory (NAT) and the Value–Belief–Norm (VBN) theory, attributes environmentally responsible behaviour to internalised moral norms and ethical values [32]. According to this view, individuals act out of a sense of personal responsibility and concern for the well-being of others and the environment, even when doing so requires personal effort or sacrifice [33,34]. The second perspective focuses on self-interest and personal benefit as the main drivers of sustainable behaviour. In this approach, pro-environmental actions are not primarily rooted in moral obligations but rather result from rational decision-making processes, where individuals engage in cost–benefit analyses. Both perspectives recognise the role of contextual and social factors in influencing sustainable behaviour and highlight the multidimensionality of pro-environmental decision-making [35]. 

To explain the behavioural intentions underlying the consumption of alternative proteins, this study adopts the Theory of Planned Behaviour (TPB), which is rooted in the second perspective. The TPB is a widely used social-psychological approach that explains individual behaviour through three key constructs, i.e., attitudes, subjective norms and perceived behavioural control [28]. It was developed to overcome the limitations of the Theory of Reasoned Action (TRA), particularly in accounting for behaviours over which individuals do not have complete volitional control. According to the TPB, behavioural intention is influenced directly or indirectly by these constructs [36,37].

To enhance the model’s explanatory power in the domain of sustainable consumption, this study adopts an extended version of the TPB, which includes two additional determinants of personal moral norms and environmental concerns. This extension is supported by the recognition that the TPB does not sufficiently account for the moral and normative dimensions of environmentally friendly behaviour. Personal moral norms reflect an individual’s internalised sense of ethical obligation—what one believes they ought to do in a given context—and thus serve for guiding behavioural standards. Similarly, environmental concerns have been shown to significantly influence both attitudes and behavioural responses related to sustainable consumption, including reduced meat intake and the adoption of alternative protein sources 

Accordingly, this study investigates the direct effects of attitudes, subjective norms, perceived behavioural control, personal moral norms and environmental concerns on consumers’ behavioural intentions towards alternative proteins. Furthermore, it examines the indirect effects of subjective norms and personal moral norms through attitudes, perceived behavioural control and environmental concerns.

Attitudes (ATT) refer to an individual’s overall evaluation—favourable or unfavourable of engaging in a specific behaviour [28]. In the context of this study, they are shaped by beliefs regarding the expected outcomes of consuming alternative protein [38]. This hypothesis is consistent with the Theory of Planned Behaviour according to which attitude is one of the main predictors of behavioural intention [28]. Studies on plant-based meat (and similar products) show that favourable attitudes towards taste, naturalness, and environmental benefits significantly increase purchase intention [39]. Attitude is also recognised as a primary driver of adoption in food innovation literature [40]. The more favourable the attitude towards alternative protein, the stronger the intention to perform the behaviour:

**H1.**  *Attitudes directly and positively affect consumer behavioural intention for alternative protein*.

Subjective norms (SNs) reflect the perceived social pressure from significant others—such as family, friends or social networks—to engage or not engage in a behaviour [28]. These norms influence not only behavioural intentions directly, but also other psychological constructs such as personal moral norms and perceived behavioural control, which, in turn, reinforce behavioural intentions. In the literature on food sustainability and alternative proteins, subjective norms play a particularly important role because the consumption of protein alternatives is often socially visible behaviour. Empirical studies show that perceived support from family, friends and social groups increases the intention to consume plant-based products [41]. Social norms can reinforce the sense of moral obligation associated with ethical and sustainable choices [42]. Accordingly, we hypothesise that:

**H2a.**  *Subjective norms directly and positively influence consumer behavioural intention for alternative protein*.

**H2b.**  *Subjective norms positively affect consumer behavioural intention for alternative protein via personal moral norms*.

**H2c.**  *Subjective norms positively affect consumer behavioural intention for alternative protein via perceived behavioural control*.

Perceived behavioural control (PBC) refers to individuals’ perceptions of their ability and autonomy to perform the behaviour [43]. Higher self-efficacy and perceived control increase the likelihood of intention formation [44] while perceived barriers decrease it [45]:

**H3.**  *Perceived behavioural control directly and positively affects consumer behavioural intention for alternative protein*.

Personal moral norms (PMNs) are internalised moral obligations that individuals use to judge whether a particular action is appropriate within a specific context [46,47]. In the domain of sustainable consumption, those with stronger personal moral norms are more inclined to act in favour of environmentally friendly alternatives. These norms may influence intention both directly, as moral drivers of action and, indirectly, by shaping attitudes and environmental concerns [8]. For instance, individuals with strong moral norms about sustainability may develop more favourable attitudes towards alternative proteins which, in turn, increases their likelihood of forming a behavioural intention [48,49]. Moral norms are an important predictor of sustainable choices [46]. The literature highlights that a strong “green” moral identity increases the intention to purchase sustainable foods [50,51]. 

Based on these premises, we formulated the following hypotheses:

**H4a.**  *Personal moral norms directly and positively affect consumer behavioural intention for alternative protein*.

**H4b.**  *Personal moral norms positively affect consumer behavioural intention for alternative protein via attitude*.

Environmental concerns (ECs) are acknowledged as a determinant of sustainable food behaviour. According to [52], both consumers and non-consumers of meat alternatives recognise the environmental benefits of such meatless products, including reduced greenhouse gas emissions, lower resource consumption and a smaller ecological footprint. However, the actual adoption of these products remains inconsistent. For occasional consumers, stronger attitudes and beliefs regarding the ecological benefits of alternative proteins may motivate the shift towards more consistent consumption. In other words, when individuals begin to associate alternative proteins with tangible environmental benefits, they may be more likely to transition from sporadic to regular consumption [53]. Accordingly, we propose the following hypothesis:

**H4c.**  *Personal moral norms positively affect consumer behavioural intention for alternative protein via environmental concerns*.

**H5.**  *High environmental concerns positively affect consumer behavioural intention for alternative protein*.

The relationship between EC and consumer behavioural intention is well supported in the literature on sustainable consumption. Environmental concerns increase the intention to reduce meat consumption and choose sustainable alternatives [54]. According to the Value–Belief–Norm Theory [32], the greater the perception of the negative impact of one’s choices on the environment, the greater the motivation to change behaviour. Numerous studies show that those who express strong environmental concerns are more likely to reduce their meat consumption or adopt alternatives, such as plant-based products or innovative proteins [55,56]. Environmental motivations are among the most consistent predictors of the intention to adopt protein alternatives, comparable to factors such as animal welfare and health impact [57]. However, the same studies show that environmental concerns often influence intention rather than actual behaviour, highlighting the attitude–behaviour gap. This makes intention a particularly appropriate variable to analyse as a direct outcome of EC. Increased environmental awareness is often correlated with a shift from sporadic to regular consumption [52].

**Figure 1:**
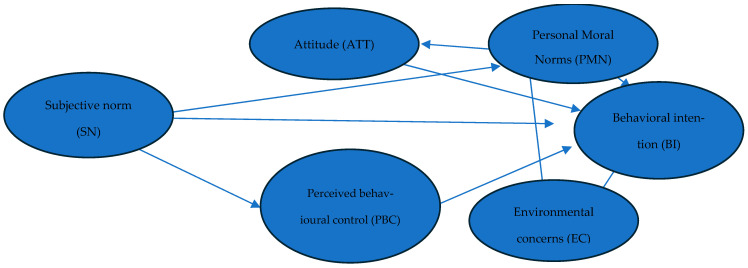
Proposed conceptual model.

Figure 1 presents the proposed conceptual model. The objective is to test whether the direct and indirect impacts of subjective norms and personal moral norms on mediating variables—attitudes, perceived behavioural control and environmental concerns—lead to stronger behavioural intentions. This is reflected in research hypotheses H2a to H2c and H4a to H4c.

**Figure 2:**
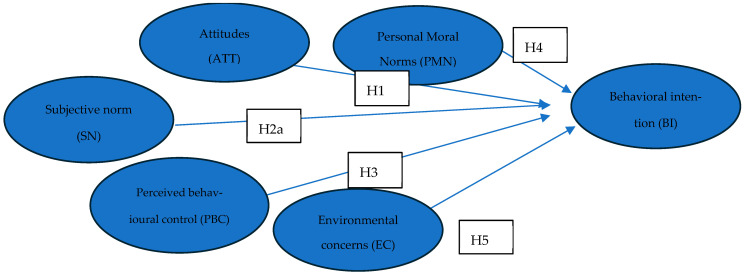
Direct effects within the proposed conceptual model.

Figure 2 illustrates the direct effects of each latent construct—attitudes (H1), subjective norms (H2a), perceived behavioural control (H3), and personal moral norms (H4a)—on consumers’ behavioural intention towards alternative proteins. It also includes the direct effect of environmental concerns on behavioural intention (H5).

**Figure 3:**
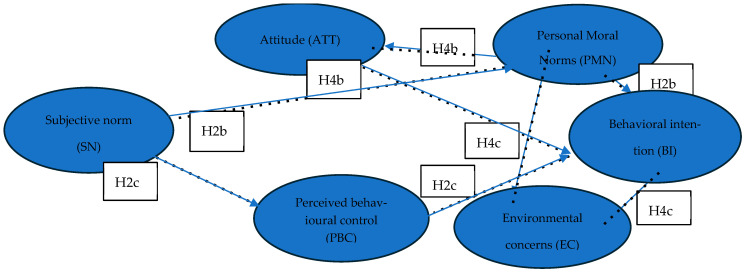
Indirect effects within the proposed conceptual model.

Figure 3 shows the indirect relationships in the model, indicating how subjective norms and personal moral norms may exert influence on behavioural intention through at least one mediating variable. Specifically, it visualises hypotheses H2b, H2c, H4b, and H4c.

## 3. Materials and Methods

### 3.1. Methodology

To test the research hypotheses derived from the extended TPB framework, we employed partial least squares structural equation modelling (PLS-SEM), which allows for the simultaneous estimation of multiple relationships between latent constructs. Each construct, typically illustrated in the model by ellipses, is a theoretical concept that is not directly observable and is operationalised through one or more manifest variables [58,59]. Latent variables can function as independent or dependent variables based on their role in the theoretical framework. Those that act only as predictors are referred to as exogenous, while those that act as outcomes or both predictors and outcomes are called endogenous.

PLS-SEM includes a structural and a measurement model. The structural model tests the hypothesised causal relationships between latent constructs by means of path analysis, based on predefined theoretical assumptions [60,61]. It examines both direct and indirect effects. Direct effects describe the influence of one construct on another without the involvement of any intermediate construct. Indirect effects occur when the influence is passed through one or more mediating constructs [62,63,64]. The measurement model explains how manifest indicators represent unobservable latent constructs [65]. Its assessment requires testing both reliability and validity. There are two ways to model the relationship between indicators and constructs. In the reflective approach, causality flows from the latent construct to the manifest indicators. In the formative approach, the indicators define the construct with each contributing a distinct aspect that collectively forms the underlying concept [66]. Reflective constructs are viewed as traits expressed through observable indicators. Removing one indicator usually does not change the overall meaning of the construct, since others still reflect the same dimension [67]. In formative models, each indicator captures distinct aspect of the construct, so omitting one alters its definition [68,69]. 

The choice of PLS-SEM is aligned with both the exploratory aims of the study and the complexity of the proposed extended TPB framework. The model integrates additional constructs—personal moral norms and environmental concerns—whose theoretical relationships are still emerging in the literature, making the analysis exploratory rather than confirmatory. In such contexts, PLS-SEM is recommended due to its prediction-oriented nature and suitability for theory development [60]. The model includes multiple latent constructs, several mediated paths and a relatively large set of indicators, conditions under which PLS-SEM performs efficiently and robustly [70]. Moreover, PLS-SEM relies on less stringent assumptions than covariance-based SEM (CB-SEM). In particular, as a non-parametric method, PLS-SEM does not rely on multivariate normality and uses resampling techniques to assess parameter significance [62]. 

All constructs are treated as reflective and assumed to be unidimensional, meaning that changes in the latent construct should lead to simultaneous variation across all indicators. This choice is consistent with the TPB framework, in which attitudes, subjective norms and perceived behavioural control are conceptualised as reflective psychological dispositions [28]. The reflective specification is also aligned with established SEM guidelines, stating that constructs representing internal psychological states should manifest through correlated indicators that co-vary as the latent trait changes [70].

The structural model expresses a linear relationship between a vector of dependent latent (endogenous) variables, denoted as η and consisting of m elements and a vector of independent latent (exogenous) variables, denoted as ξ:

η = Βη + Γξ + ζ(1)

where B is the matrix of regression coefficients representing the relationships among the endogenous latent variables, Γ is the matrix of regression coefficients that link the endogenous variables to the exogenous ones (ξ), and ζ is the vector of disturbance terms, which are assumed to be normally distributed with a mean of zero and uncorrelated with the exogenous latent variables.

The measurement model specifies how the latent constructs are related to their manifest indicators: This applies to both endogenous (2) and exogenous (3) variables, as shown below:

y = Λ_y_η + ε (2)

x = Λ_x_ξ + δ (3)

where y and x are the vectors of manifest indicators, Λ_y_ and Λ_x_ are the matrices of the regression coefficients that relate y to η and x to ξ, respectively, and ε and δ are the corresponding vectors of errors.

The evaluation of the measurement model and the explanatory capacity of the structural model was conducted according to three main criteria: 

Item reliability was assessed using factor loadings, considered acceptable if ≥0.40 and preferably ≥0.70 [62]. Items below this threshold should be removed to ensure a well-fitting model [71]. 

Internal consistency was measured by Cronbach’s alpha, with recommended values between 0.70 and 0.90 [72], and with composite reliability (CR), which represents a more accurate indicator in PLS-SEM contexts [73]. A CR ≥ 0.70 indicates good representativeness of the indicators [74]. 

Convergent validity was estimated by the average variance extracted (AVE), defined as the average of the squared weights of the indicators associated with a construct. A value ≥0.50 indicates that the construct explains more than half of the variance of its indicators [75]. 

### 3.2. Data

The extended TPB framework was empirically tested using data collected through a questionnaire entitled “Survey on Nutrition and Health, Environmental Sustainability and Attitudes towards Innovative and Sustainable Products”. The survey involved 948 individuals residing in the Campania region (southern Italy) and was designed to assess respondents’ willingness to consume alternative protein products as part of broader efforts to promote environmentally sustainable food choices.

Data collection was carried out over a six-month period, from February to July 2024, using the Computer-Assisted Web Interviewing (CAWI) methodology. To preserve respondent anonymity and ensure efficiency in administration, the questionnaire was distributed electronically. Participants were contacted via email, informed of the study’s purpose and invited to complete the survey through a personalised link. The decision to adopt an online format was based on its advantages in terms of rapid deployment, flexibility, cost efficiency and the elimination of manual data entry [76]. All respondents completed the questionnaire in its entirety. Prior to launching the full survey, a pre-test was conducted on a small group to verify the clarity, relevance and interpretability of the questions, and the survey’s overall quality. As the research involves human participants, it was performed in compliance with ethical guidelines and ensured that the privacy rights of participants were fully observed. The study was completely anonymous, and minors were not included. Informed consent was obtained from all participants. 

The questionnaire was developed to capture (i) demographic characteristics (age, gender, marital status, education, occupation, household composition and income); (ii) dietary habits, health-related behaviours, and food safety or sustainability concerns, with attention to adherence to specific dietary regimes (e.g., vegan, paleo, vegetarian, gluten-free), motivations for these choices (e.g., weight loss, diabetes, allergies), and experiences with eating disorders (e.g., anorexia, bulimia, binge eating); (iii) food purchasing behaviour, focusing on decision-making criteria (e.g., packaging, environmental certification, product origin, nutritional information, brand and price); (iv) consumption patterns for protein-rich (e.g., meat, eggs, dairy, legumes) and other food categories (e.g., cereals, fruits, and vegetables); (v) behavioural intentions towards alternative proteins, together with a range of psychological constructs (e.g., concerns, pro-environmental behaviour, attitudes, subjective norms, perceived behavioural control, personal moral norms, awareness, attribution of responsibility, opinions, and beliefs).

Based on [21], who analysed consumer attitudes towards alternative protein sources in Germany, this survey explores participants’ willingness to include three categories of meat alternatives—i.e., insects, cultured meat and plant-based meat—into their diets to support environmental sustainability. Respondents were asked to indicate how often they would be willing to consume these products every week, using a scale from 0 (never) to 7 (every day). The questionnaire also assessed awareness of key environmental issues (e.g., climate change, public health risks, and the link between dietary choices and sustainability). In addition, it explored personal moral norms related to the purchase of alternative proteins, perceived behavioural control over purchasing decisions and attitudes towards societal norms concerning sustainable eating.

Participation in the survey did not follow a probabilistic sampling strategy. Consequently, the socio-demographic composition of the respondents does not fully reflect the population structure of the region, and the sample should not be considered statistically representative. However, this does not undermine the aims of the study. Given its exploratory nature and the use of PLS-SEM, statistical representativeness is not required for valid structural estimates. PLS-SEM is prediction-oriented and theory-building and its inferential robustness derives from adequate sample size, the reliability of the measurement model and the stability of bootstrap procedures rather than probabilistic sampling. In this regard, the large sample size and the strong reliability and validity metrics obtained support the stability and interpretability of the estimated relationships. The required sample size was determined in advance based on an 80 per cent statistical power threshold [77] and Cohens’ effect-size benchmarks [78] (0.20, 0.50, and 0.80 for small, medium, and large effects, respectively). The final sample size of 948 respondents approaches the threshold for detecting small effects (size requirement = 1362) and exceeds that required for medium effects (size requirement = 304). The study is therefore adequately powered to detect medium and large effects within the PLS-SEM framework.

Table 1 shows the latent constructs (Column 1), their labels (Column 2), and the meaning of the manifest indicators (Column 3) used for each construct. All items were measured using a 5-point Likert scale, ranging from 1 = “strongly disagree” to 5 = “strongly agree”. The table is complemented by descriptive statistics in the last two columns.

The attitude (ATT) construct, based on [28,79], reflects a generally positive perception of alternative protein product consumption. The average scores for the three items show that participants share attitudes quite uniformly. The perceived behavioural control (PBC) construct captures individual’s perceived ability to make informed food choices. For subjective norms (SNs), the highest average score in item sn1 reflects perceived expectations from others regarding sustainable protein consumption. Item sn4, concerning policy incentives, shows a slightly lower but still relevant value (0.728), suggesting openness to institutional support. The personal moral norm (PMN) construct records the highest average values (up to 0.944), confirming the presence of a strong sense of moral responsibility towards sustainable consumption. The consistency between the items, combined with low standard deviations, suggests a shared and stable belief among participants.

Similarly, the environmental concern (EC) construct shows consistently high average scores (from 0.814 to 0.946) and low variability, confirming the importance attributed to environmental sustainability. The item concerning the link between environmental protection and personal health (ec3) stands out with the highest average. Finally, the behavioural intention (BI) construct highlights consumers’ willingness to include alternative proteins in their diets. Intentions are stronger for cultured meat (bi2, mean = 0.882) and plant-based products (bi3, mean = 0.809), while willingness to consume insect-based protein is slightly lower (bi1, mean = 0.711). This could suggest greater cultural acceptability for certain alternative protein sources. Behavioural intention was modelled as a single reflective construct capturing consumers’ overall intention to adopt alternative proteins. Although the three indicators refer to three different categories of protein sources (i.e., insects, cultured meat and plant-based proteins), they reflect the same underlying behavioural domain—namely, the willingness to include alternative proteins in one’s diet.

Overall, the descriptive statistics show high internal consistency within each construct and reflect a generally positive orientation towards sustainable dietary practices, motivated by personal attitudes, moral and environmental norms and social expectations.

## 4. Results

To test the research hypotheses outlined in Section 2, PLS-SEM was applied using a non-parametric bootstrap procedure with 5000 resamples, allowing for the estimation of standard errors and the assessment of the statistical significance of model parameters [62]. The results presented in the following sections cover the assessment of the measurement model and the main findings of the structural model.

### 4.1. Measurement Model Assessment

The measurement model was built using an iterative process, whereby indicators were added or removed within each construct to establish a strong relationship between the manifest indicators and their respective constructs. Given the reflective nature, multicollinearity does not represent a concern.

Table 2 shows the factor loadings, which indicate the extent to which each indicator reflects its corresponding latent variable. All loadings exceed the recommended threshold of 0.70, with many above 0.8 or even 0.9, indicating excellent indicator reliability. This suggests that the indicators strongly represent their respective latent variables and, in line with the criteria proposed by [60], the requirement for convergent validity is fully satisfied.

### 4.2. Structural Model

Table 3 presents the reliability and validity statistics for all constructs, together with the explanatory capacity of the structural model. Internal consistency was evaluated through Cronbach’s alpha and composite reliability (rho_a and rho_c). All constructs exceeded the recommended threshold of 0.70, confirming high internal reliability. Convergent validity, measured by the average variance extracted (AVE), was also satisfactory for all constructs, with values above the 0.50 benchmark. The model’s explanatory capacity was evaluated primarily through the adjusted R^2^ coefficients, which account for the number of predictors and penalise unnecessary model complexity. Subjective norms exert the largest effects: a medium-to-high effect on personal moral norm (≈0.20–0.25) and a medium effect on perceived behavioural control (≈0.15–0.20). These results align with the Theory of Planned Behaviour, in which social pressure and perceived expectations often shape both moral obligations and perceived control over behaviour. The influence of personal moral norm on attitude, behavioural intention, and environmental concerns was small-to-medium (0.06–0.10). Overall, the reliability and validity diagnostics confirm that the measurement model is robust, with strong internal consistency and convergent validity. The structural results suggest statistically significant relationships of moderate magnitude, consistent with the latent, psychological nature of the constructs.

Table 4 presents the path coefficients and corresponding significance levels for the structural model estimated using the PLS-SEM method. The analysis examines the relationships among the main latent variables, i.e., attitudes (ATT), behavioural intention (BI), personal moral norms (PMNs), subjective norms (SNs), perceived behavioural control (PBC) and environmental concerns (ECs).

In summary, the model confirms only some of the relationships that have been hypothesised: personal moral norms and subjective norms are the main predictors within the proposed conceptual framework. Specifically, the model for single hypotheses showed that:

**H1.** *Attitudes directly and positively affect consumer behavioural intention for alternative protein*.

Despite the generally positive attitudes towards alternative protein consumption, such attitudes do not translate into stronger intentions (β = 0.037; *p* = 0.793), highlighting the often-cited intention–behaviour gap. This pattern is common in sustainable food research, where people report positive attitudes towards ethical/green products, but these attitudes do not directly predict intention or purchase [44]. This is because contextual factors (price, availability, perceived taste, convenience) outweigh the overall evaluation of the product. When other ethical or normative constructs are strong (such as PMN), attitude can become a weak predictor. The literature shows that in morally charged behaviours, personal norms outperform attitudes as predictors [92].

**H2a.** *Subjective norms directly and positively influence consumer behavioural intention for alternative protein*.

The direct effect of subjective norms on behavioural intention for alternative protein is not statistically significant, indicating that social influence operates less as external pressure and more as a normative and motivational support structure. Subjective norms strongly influence BI via PMN and PBC (both highly significant). This pattern is common, as SN acts more as an antecedent construct than a direct predictor. The literature on TPB indicates that in ethical choices, subjective norms rarely directly predict intention, but do so indirectly through personal norms [42,93].

**H2b.** *Subjective norms positively affect consumer behavioural intention for alternative protein via personal moral norms*.

Subjective norms to personal moral norm (PMN) show a medium-to-high effect, indicating that subjective norms significantly influence perceived moral obligation. On the other hand, subjective norms play a predominantly indirect role. They directly shape personal moral norms (*β* = 0.469; *p* < 0.001), reinforcing the internalisation of ethical standards and simultaneously enhance perceived behavioural control (PBC) (*β* = 0.416; *p* = 0.007), supporting individuals’ sense of autonomy in making sustainable choices. However, their direct influence on behavioural intention is not statistically significant (*β* = 0.181; *p* = 0.165), indicating that social influence operates less as external pressure and more as a normative and motivational support structure.

Regarding the indirect effects, the path SN -> PMN -> BI is highly significant (*p* < 0.001), with a positive path coefficient of 0.469. This suggests that social norms (SN) have a positive effect on behavioural intention (BI) through the influence of PMN (possibly a variable such as personal or moral norms). The high t-statistic value (5.710) indicates that the effect is not random and the very low *p*-value (0.000) supports the significance of this indirect effect. This path indicates that social norm perceptions indirectly influence behavioural intentions, suggesting the importance of social influences on individual choices.

**H2c.** *Subjective norms positively affect consumer behavioural intention for alternative protein via perceived behavioural control*.

Subjective norms to perceived behavioural control (PBC) show a medium effect, suggesting a meaningful yet more moderate impact. Furthermore, subjective norms also have a significant impact on perceived behavioural control, emphasising the role of social references not only in moral formation but also in shaping individuals’ confidence in their ability to act sustainably. The coefficient of 0.416 suggests that greater social norm influence increases behavioural intention through a positive perception of personal control. As in the previous case, the *p*-value of 0.000 and the high t-statistic (5.199) confirm that this effect is statistically significant. This result implies that perceptions of control, influenced by social norms, play a crucial role in determining the intention to perform a behaviour, highlighting a mediation process between social influences and individual decisions.

**H3.** *Perceived behavioural control directly and positively affects consumer behavioural intention for alternative protein*.

The direct effect of perceived behavioural control (PBC) on behavioural intention (BI) is not statistically significant. The *p*-value > 0.05 and the very low t-score indicate that people do not perceive their control over actions as a determining factor in the intention to consume alternative proteins. PBC does not directly influence intention, but SN -> PBC -> BI is a very significant indirect effect. This is because PBC is often insignificant for readily available products. The literature shows that when a behaviour is perceived as easy to perform or does not require particular effort, PBC loses its predictive power [36,94].

**H4a.** *Personal moral norms directly and positively affect consumer behavioural intention for alternative protein*.

Personal moral norm as a predictor (towards ATT, BI and EC) show small-to-medium effects. This suggests that PMN plays a significant but not dominant role in explaining the variance of the dependent constructs.

Personal moral norms exhibit a significant triple influence on behavioural intention: (i) they positively affect attitude towards sustainable consumption (*β* = 0.268; *p* = 0.014); (ii) have a direct impact on behavioural intention (*β* = 0.234; *p* = 0.043), and (iii) contribute to strengthening environmental concerns (*β* = 0.281; *p* = 0.007). These findings align with recent literature [95], suggesting that internalised moral beliefs are a crucial driver of pro-environmental behaviour.

**H4b.** *Personal moral norms positively affect consumer behavioural intention for alternative protein via attitude*.

The indirect effect PMN -> ATT -> BI suggests that personal norms (PMN) influence behavioural intention (BI) through a change in attitudes. The coefficient of 0.268 implies that greater adherence to personal or moral norms strengthens favourable attitudes, which in turn increases the intention to act. With a t-statistic of 2.456 and a *p*-value of 0.014, this effect is significant, although weaker than the previous paths. This path highlights how personal norms can enhance attitudes towards a behaviour, which then increases the intention to engage in actions that align with those norms.

**H4c.** *Personal moral norms positively affect consumer behavioural intention for alternative protein via environmental concerns*.

The path PMN -> EC -> BI suggests that personal norms (PMNs) influence behavioural intention (BI) through environmental concern (EC). This implies that personal or moral norms influence behaviour, even mediated by the ability to understand and share the emotions of others. The path coefficient of 0.281 and the t-statistic of 2.676, along with a *p*-value of 0.007, indicate that this effect is significant and that empathy plays an important role in determining behavioural intentions. This path suggests that the ability to empathise with others, influenced by personal norms, can be a key factor in influencing the intention to engage in certain behaviours. The indirect effects show that both social norms and personal norms impact behavioural intention (BI) not just directly, but also through various mediators such as perceived control, attitudes and empathy. All indirect effects are significant, with *p*-values ranging from 0.000 to 0.014, indicating that these mediated paths are robust and contribute significantly to understanding the factors that influence behavioural intention. In general, the results suggest that social and personal norms influence behavioural intentions not only directly, but also through a range of psychological and social mechanisms, such as control perceptions, attitudes, and empathy.

**H5.** *High environmental concerns positively affect consumer behavioural intention for alternative protein*.

In contrast to the TPB expectations, attitude, perceived behavioural control, and environmental concerns do not directly influence behavioural intention. Despite the generally positive attitudes towards alternative protein consumption, such attitudes do not translate into stronger intentions (*β* = 0.037; *p* = 0.793), highlighting the often-cited intention–behaviour gap. Similarly, although environmental concern is generally high among respondents, it does not independently drive intention (*β* = 0.041; *p* = 0.720), implying that environmental concern alone is insufficient to motivate behavioural change. The same applies to perceived behavioural control, which also shows no direct effect (*β* = 0.048; *p* = 0.635). This result is very common in the literature on plant-based eating. Many studies show that EC does not directly predict intention, but acts on other psychological variables (attitude, PMN), which then influence intention [40,53]. This is exactly what emerges in our model: PMN -> EC -> BI is significant, but EC -> BI direct is not. EC is a weak predictor in behaviours with a high hedonic component, as food choices are guided by taste, not environmental values [96]. Environmental concerns are often not enough to translate into intention unless supported by sensory value. Many consumers report having strong environmental concerns but do not change their purchasing intentions [44]. The consumption of protein alternatives is particularly influenced by the “hedonic gap”: people believe that the product is less tasty.

The findings indicate that personal moral norms (PMNs) exert a stronger influence on behavioural intentions than the classical TPB constructs of attitude, subjective norms and perceived behavioural control. This result is consistent with a long tradition of research showing that moral self-regulation can become a primary determinant of prosocial and pro-environmental behaviours when actions are perceived as ethically significant [97]. Several studies have shown that personal moral obligations can outperform or complement TPB predictors, especially in areas involving moral considerations, such as environmental choices, sustainable consumption or ethical decision-making [98]. These results thus reinforce the growing consensus that the TPB may underrepresent moral influences and that including PMN is essential for capturing the motivational dynamics underlying ethically salient behaviours.

## 5. Discussion

The results of this study highlight the fundamental role of Personal Moral Norms (PMNs) in determining behavioural intention (BI) in the context of sustainable food choices. PMNs directly influence intention while also mediating the effects of subjective norms (SNs) and environmental concerns (ECs). This result shows remarkable consistency with extended behavioural models that integrate moral dimensions [32,46,99]. 

### 5.1. Cognitive Barriers and Intention–Behaviour Gaps in Innovative Food Choices

The analysis of the results for hypotheses highlights interesting results. The first hypothesis*—attitudes directly and positively affect consumer behavioural intention for alternative protein* (H1)—shows that the lack of a significant direct relationship between classical predictors such as attitude (ATT), perceived behavioural control (PBC), and BI (as suggested by the TPB methodological approach) leads to the argument that internalised moral values may have greater explanatory power in guiding environmentally sustainable food behaviours presumably associated with innovative or sustainable agricultural practices (e.g., alternative or plant-based protein sources). The “intention–behaviour gap” in the case of low-impact protein alternatives is a phenomenon confirmed by the main reference in the literature on sustainable consumption, which highlights how consumers, despite declaring favourable attitudes and positive intentions, are often inconsistent with their purchasing choices. This is due to several factors. Product perception plays a significant role; consider, for example, food neophobia, which manifests itself in a distrust of new foods or those perceived as unsuitable for consumption, such as innovative plant-based proteins or cultured meat and insects. Added to this is often the belief that the taste or appearance are not up to the level of conventional meat and unfamiliarity, linked to lack of direct experience, reduces trust [48,52]. Other factors may include prices, considered by many to be too high, and poor availability in usual outlets [57,100]. Furthermore, there are social aspects related to one’s dietary identity that make traditional meat consumption perceived as normal [101,102]. Finally, contextual factors must be taken into account, especially those related to lack of time, a lack of habit of cooking protein alternatives, and poor visibility in supermarkets [56]. 

In relation to the third hypothesis—*perceived behavioural control directly and positively affects consumer behavioural intention for alternative protein* (H3)—the results show that the perceived behavioural control (PBC) neither has a significant effect nor interacts as expected with attitudes and subjective norms, on consumer behaviour. This suggests that often the relationship between PBC and intention does not materialise as in the classic model. In contexts of food innovation, such as alternative proteins, where external factors such as limited availability, high price or lack of knowledge on how to prepare them often have a significant impact, and where purchases are often influenced more by emotional, moral or social factors, the perception of control may be less relevant because purchases are often influenced more by emotional, moral, or social factors rather than by the simple perceived ability to act. Therefore, uncertainties arising from product novelty may limit the direct effect of PBC [40,103].

Under these conditions, favourable attitudes do not automatically translate into intentions, because they are not yet supported by concrete experiences, repeated consumption practices, or sufficient product availability. In essence, the uncertainty and unfamiliarity that characterise alternative proteins foster psychological barriers, perceived risk and neophobia. Therefore, the non-significance in this case is not an anomaly but rather confirms the limitations of rational models when dealing with novel or culturally distant foods. Similarly, perceived behavioural control may be weak when the target behaviour is poorly understood or perceived as difficult to integrate into one’s eating habits, as occurs in diets characterised by a strong culinary tradition. Therefore, the non-significance of ATT and PBC observed in this study appears consistent with the literature investigating emerging eating behaviours, where cultural, psychological, and contextual barriers tend to play a dominant role.

### 5.2. The Role of Social Influence and Internalisation Mechanisms

Analysing hypothesis (H2a)—*subjective norms directly and positively influence consumer behavioural intention for alternative protein*—it emerges that even subjective norms such as attitudes do not exert a significant direct influence on behavioural intention towards alternative proteins. This indicates, according to [104], that social influence operates less as external pressure and more as a normative and motivational support structure. When studying innovative phenomena such as alternative protein consumption, subjective norms often do not directly influence purchase intention. When meat consumption is culturally embedded, subjective norms related to family and friends’ expectations may be weak or perceived as marginal and fail to influence intentions [38]. The literature on the topic often highlights that for innovative choices such as alternative proteins, personal attitudes (e.g., beliefs about health, taste and sustainability) and perceived behavioural control weigh more heavily than subjective norms. Norms may play an indirect role, acting through the reinforcement of favourable attitudes, but they do not directly influence intention [100,105]. In reality, alternative proteins are still perceived as niche behaviours, and the lack of strong social cues reduces the power of subjective norms to drive intentions [56,106]. According to several studies on sustainable products, subjective norms influence intention only through personal beliefs or moral norms (personal norms), and not directly.

The other hypothesis on subjective norms (H2b)*—subjective norms positively affect consumer behavioural intention for alternative protein via personal moral norms*—shows a strong overall effect on personal moral norms (PMN) which suggest that social influence plays a key role in shaping internalised moral obligations. This confirms previous scientific studies [32,42] on the transformation of external normative pressure into internal moral commitments. Overall, the results suggest that behavioural intention is less driven by cognitive or control-related factors and more by normative and internalised dimensions, such as moral conviction and social context. In this perspective, personal moral norms emerge as a key mediating variable, bridging the influence of social norms with internal dispositions like attitudes and environmental concerns.

When subjective norms, which represent the expectations of relevant people, favour the consumption of alternative proteins, they can be internalised by the consumer as personal moral norms. This transforms external social pressure into an internal ethical obligation, consistent with values such as environmental sustainability or animal welfare [38]. Subjective norms influence moral norms which, in turn, guide intentions. For innovative products, this is important since the choice is often motivated by ethical reasons. In this case, addressing moral norms likely helps reduce the conflict between the habit of eating traditional meat and ethical beliefs [56].

An interesting aspect to understand is if *subjective norms positively affect consumer behavioural intention for alternative protein via perceived behavioural control* (H2c). Our study confirms that personal moral norms represent a hallmark of the responsible consumer, who bases his or her purchasing decisions not only on functional criteria such as price and utility, but also on ethical, environmental and social values in accordance with [107,108]. If family and friends’ expectations are favourable towards alternative protein consumption, then they can strengthen consumers’ confidence in their ability to implement the behaviour, increasing perceived behavioural control (PBC) [106]. This phenomenon is particularly important for innovative products such as alternative proteins where, as has been seen, the perception of difficulties related, for example, to cultural and psychological aspects, availability and price represent a significant obstacle. Indeed, the support of one’s social reference group can reduce the perception of obstacles and make the behaviour appear more easily achievable. Therefore, the combined effect of subjective norms and PBC significantly strengthens purchase intention. The role of social norms and their internalisation must also be interpreted in light of the highly culturally specific context under analysis. The Campania region, like other regions of southern Italy, is characterised by a deeply rooted gastronomic heritage and a traditional Mediterranean diet centred on typical local products. In such contexts, cultural and familial norms related to food exert a strong influence, both in guiding food preferences and in defining what is considered socially acceptable or “normal” to consume. This can constitute a barrier to the acceptance of products perceived as distant from tradition, such as alternative proteins.

### 5.3. Personal Moral Norms and Intentions Towards Alternative Proteins

An important role in the results of this study is attributed to personal moral norms. Specifically, *personal moral norms directly and positively affect consumer behavioural intention for alternative protein* (H4a). Some recent empirical studies confirm the role of personal moral norms in shaping pro-environmental and pro-sustainable intention and behaviour, particularly in food contexts. Personal norms have been identified as predictors of the intention to choose sustainable meals and as the most influential factor in sustainable food consumption decisions, with several studies showing that reduction in consumption are driven by specific underlying psychological process [31,109]. This process includes values, awareness of consequences, attribution of responsibility, and finally the activation of personal moral norms that guide action. According to [42], it can be argued that personal norms explain pro-environmental behaviours even beyond the classic TPB constructs, such as subjective attitudes and norms, due to their deep-rooted connection with internalised values and awareness of moral consequences [32,42]. In the context of alternative proteins, personal moral norms reflect ethical commitment to environmental sustainability, health and animal welfare. When a consumer perceives a personal moral obligation to reduce their consumption of traditional meat, their intention to choose alternative proteins is strengthened. In this sense, personal moral norms help increase the consistency between what the consumer believes is right and what they actually do, thus directly increasing the intention to adopt alternative proteins [110]. Recent studies highlight how personal moral norms are among the strongest predictors of the intention to consume alternative proteins more than attitudes or subjective norms, precisely because they involve a sense of individual ethical responsibility [52,56]. 

In addition, *personal moral norms positively affect consumer behavioural intention for alternative protein via attitude* (H4b). According to [111], moral decisions strongly support the idea that personal norms can influence behaviour through attitude changes. In particular, moral norms can significantly alter moral choices, justifying the importance of the norm as a mediator of attitude. In the case of alternative proteins, personal moral norms may underlie a positive attitude towards sustainable consumption, as the behaviour is perceived as morally appropriate [112]. Moral norms can reinforce a favourable attitude towards alternative proteins, which in turn increases purchase intentions towards alternative protein consumption [52]. 

Furthermore, *personal moral norms positively affect consumer behavioural intention for alternative protein via environmental concerns* (H4c). PMNs significantly influence environmental concerns, indicating that individuals with stronger moral convictions are more likely to express greater environmental awareness. Furthermore, subjective norms also have a significant impact on perceived behavioural control, emphasising the role of social references not only in moral formation but also in shaping individuals’ confidence in their ability to act sustainably. Personal moral norms represent a hallmark of the responsible consumer, who bases his or her purchasing decisions not only on functional criteria such as price and utility, but also on ethical, environmental and social values in accordance with [107,108]. This new and more evolved consumer profile reflects a deeper sense of responsibility towards the broader consequences of consumption, such as environmental degradation, animal welfare or social justice within the supply chain. Unlike external pressures or social expectations, PMNs are rooted in internalised moral obligations and are therefore understood as self-imposed duties that reflect a strong internal commitment to “doing the right thing.” Examples of this reasoning include choosing sustainably produced foods or avoiding products with a negative environmental impact, establishing a stable basis for consistent pro-environmental and pro-social behaviour in accordance with [34]. Personal moral norms refer precisely to internalised moral obligations that drive the individual to act in line with ethical principles, even in the absence of external pressures. According to [98], they represent the internal engine that transforms values into action, overcoming the effectiveness of classic TPB variables such as attitude and perceived control. PMNs transform abstract values such as sustainability, justice or compassion into behavioural intentions and concrete actions. This transformation gives PMNs greater explanatory power than traditional components of the Theory of Planned Behaviour (TPB), such as attitude or perceived behavioural control. In fact, attitudes may reflect a favourable opinion and PBC a sense of feasibility, but neither necessarily implies a moral obligation. PMNs, on the other hand, introduce a psychological dimension, a sense of duty, which strengthens the link between values and action and increases behavioural consistency even in conditions of discomfort or personal cost. Furthermore, when placed in consumer contexts such as food choices, PMNs become particularly relevant. According to [44], they help explain why some individuals consistently choose plant-based, fair trade or locally produced options, despite higher costs or lower availability. In conclusion, this internalised moral compass distinguishes the responsible consumer not only by what they buy, but also by why they buy it, revealing consumption as a form of ethical expression and civic participation [107].

In the case of alternative proteins, personal moral norms may underlie a positive attitude towards sustainable consumption, as the behaviour is perceived as morally appropriate [112]. Moral norms can reinforce a favourable attitude towards alternative proteins, which in turn increases purchase intentions towards alternative protein consumption [52,56]. 

### 5.4. The Influence of Environmental Concerns on Innovative Food Choices

The analysis of the last hypothesis draws attention to a very important topic by verifying whether *high environmental concerns positively affect consumer behavioural intention for alternative protein* (H5). Contrary to many studies that report a positive influence of environmental concern (EC) on pro-environmental intentions, there can also be a lack of significant direct impact in some contexts. Even if consumers express high levels of environmental concern, this does not always translate into a positive intention towards alternative proteins due to the difference between concerns and actual behaviour, which can be referred to as the “intention–behaviour gap.” As seen above, this gap may depend on ingrained habits, lack of accessible alternatives or perceived barriers that prevent concerns from being transformed into alternative protein consumption behaviour [57]. Furthermore, purchase intentions are often more influenced by other factors such as taste, cultural habits, price and convenience. In fact, a portion of environmentally conscious consumers may not yet perceive sufficient immediate personal or social benefits in switching to alternative proteins, thus reducing the direct effect of environmental concerns on intention [56]. 

Taken together, the results indicate that moral and social dimensions outweigh purely attitudinal or control-related factors in predicting behavioural intentions towards alternative proteins. While these findings highlight the psychological mechanisms underlying sustainable food choice, it is important to acknowledge that the study relies on self-reported measures of behavioural intention, which are subject to well-documented biases such as social desirability, moral signalling and the overstatement of pro-environmental intentions [113,114]. These biases are common in sustainability-related research, where stated intentions often reflect socially valued norms rather than actual behavioural tendencies [115,116]. As a result, reported intentions may only partially capture real consumption behaviour, reinforcing the well-known intention–behaviour gap observed in ethical and sustainable food domains [101,117]. Although this does not undermine the internal validity of the model, it calls for caution when interpreting effect magnitudes and underlines the need for future research to complement self-reported data with behavioural or experimental evidence [118]. 

This study has some limitations. First, the analysis does not differentiate behavioural intentions among the three alternative protein categories considered (plant-based, animal-based, and non-animal-based), which may present distinct psychological and perceptual factors. Second, the data are based on self-reported measures, which are inherently subject to social desirability and hypothetical biases, particularly in areas related to sustainability and ethical consumption. Third, the sample is limited to residents of the Campania region, an area characterised by a strong culinary tradition.

## 6. Conclusions and Policy Implications

This study highlights the central role of personal moral norms, which directly influence consumers’ intentions to purchase alternative protein foods by activating a sense of individual ethical responsibility. When consumers perceive a moral obligation to reduce their consumption of traditional meat, their intention to choose alternative proteins increases. Personal moral norms can also reinforce favourable attitudes towards alternative proteins, which in turn strengthen purchase intentions. Moreover, individuals with strong personal moral norms related to sustainability tend to express greater concern for the environmental impact of their food choices, thereby encouraging the adoption of sustainable alternative protein sources. For innovative products, addressing moral norms helps reduce the conflict between traditional meat consumption habits and ethical beliefs. When consumers receive support from their social reference group, they may perceive fewer obstacles to adopting these products and perceive the behaviour as more easily achievable.

On the other hand, subjective norms often do not directly influence purchase intentions in contexts where meat consumption is culturally embedded. Likewise, environmental concerns do not always translate into positive intentions towards alternative proteins due to the well-documented gap between concern and behaviour. Environmentally conscious consumers may lack awareness of the personal benefits associated with switching to alternative proteins, reducing the direct impact of environmental concerns on intention. This inconsistency in purchasing behaviour is also shaped by product perceptions, unfamiliarity, price and food culture. Finally, for innovative products such as alternative protein foods, emotional, moral and social factors tend to outweigh perceived behavioural control.

The findings highlight the importance of regional specificities and cultural identity in shaping the propensity for sustainable dietary innovation. The promotion of alternative protein foods requires not only greater market accessibility but also communication strategies that emphasise ethical values and sustainability. Effective policies should therefore move beyond traditional information tools and prioritise the activation of moral commitment and prosocial values. From this perspective, impactful strategies might include storytelling, emotional appeals and campaigns that emphasise ethical responsibility, environmental protection and intergenerational equity. Institutions also play a key role by supporting subsidies for sustainable products and by promoting sustainability education in schools. Stronger integration of sustainability goals, combined with attention to moral and cultural identity, can enhance both public and private efforts to foster the transition towards more sustainable food models. Ultimately, fostering the adoption of alternative proteins in culturally rich regions like Campania requires more than information provision. It demands cultivating moral commitment and transforming sustainable choices into shared social norms. 

According to the study’s findings, personal moral norms are a primary determinant of the intention to adopt alternative proteins. Therefore, policies should incorporate communication strategies that combine persuasive narratives, emotional campaigns and educational initiatives aimed at highlighting both the environmental and personal and collective benefits associated with the protein transition. Public policies should emphasise the personal and collective benefits of the transition, highlighting advantages such as health, food security and the resilience of production systems. They should also seek to promote sustainable food innovation through social leverage, for example, by promoting initiatives with schools and universities. At the same time, public policymakers could intervene to reduce economic and market barriers through targeted incentives or subsidies to reduce the price of sustainable products. Furthermore, to reduce the conflict between tradition and innovation, since cultural identity and territorial specificities influence the propensity for change, policies sensitive to the territorial context are essential, especially in areas with a strong gastronomic tradition such as the Campania region. Finally, it must be taken into account that transforming food systems requires a multi-stakeholder approach aimed at encouraging collaboration with the private sector to increase the supply and quality of alternative proteins, as well as the commitment of local agri-food supply chains.

Future research could examine the moderating role of socio-demographic characteristics (e.g., through multigroup PLS analysis) to identify potential heterogeneity in psychological drivers across consumer segments. In addition, future studies may analyse behavioural intentions separately for the three categories of alternative proteins—insects, cultured meat and plant-based meat—as specific responses may emerge. Category-specific models could extend the understanding of behavioural mechanisms not explored in the present study. The study design could also be expanded to consider a national sample, which could provide a deeper understanding of psychological drivers and environmental and socioeconomic dynamics that differentiate these protein sources.

## Figures and Tables

**Table 1:** Latent constructs, manifest indicators, and items. Descriptive statistics.

Latent Construct	Item	Label	Mean	St. Dev (SD)
Attitude (ATT) [28,79]	It is important to consume protein products	att1	0.801	0.131
	Consuming protein products is important for our health	att2	0.791	0.165
	Choosing protein products can affect greenhouse gas emissions	att3	0.828	0.130
Perceived Behavioural Control (PBC) [28,80]	I believe that I have the knowledge to choose between different protein products	pbc1	0.685	0.142
	Choosing which protein products to buy is entirely up to me	pbc2	0.698	0.096
	I have the resources to be able to choose which protein products to buy	pbc3	0.731	0.112
	I know the difference between different protein products	pbc4	0.731	0.112
Subjective Norms (SN) [28,81,82]	I am expected to buy sustainable protein products for the improvement of environmental quality	sn1	0.873	0.032
	People significant to me buy sustainable protein products	sn2	0.807	0.060
	People significant to me would advise me to consume more sustainable protein products every day	sn3	0.840	0.038
	If the local government provided subsidies, I would like to buy sustainable protein products	sn4	0.728	0.070
Personal Moral Norm (PMN) [83,84,85]	I believe I have a moral obligation to purchase sustainable alternative protein products	pmn1	0.914	0.020
	I would feel guilty if I did not purchase sustainable alternative protein products	pmn2	0.855	0.037
	Purchasing sustainable alternative protein products is in line with my principles of environmental protection	pmn3	0.944	0.010
Environmental Concerns (EC) [86,87,88,89,90].	Climate change reduces the chances of survival of humans and animal and plant species	ec1	0.814	0.076
	Environmental protection promotes my health	ec2	0.946	0.037
	Environmental protection improves the quality of my life	ec3	0.926	0.039
	Human-caused environmental damage today affects the well-being of future generations	ec4	0.892	0.059
Behavioural Intention (BI) [91]	For greater environmental sustainability how often weekly would you be willing to consume alternative proteins such as insects	bi1	0.711	0.128
	For greater environmental sustainability how often per week would you be willing to consume alternative proteins such as cultured (synthetic) meat	bi2	0.882	0.061
	For greater environmental sustainability with what weekly frequency would you be willing to consume alternative proteins such as plant-based meat	bi3	0.809	0.086

**Table 2:** Individual item reliability: Factor loadings.

Latent Construct	Item	Factor Loadings	St. Dev	*t*-Statistic	*p*-Value
Attitude (ATT)	att1	0.828	0.131	6.340	0.000
	att2	0.826	0.165	5.008	0.000
	att3	0.843	0.13	6.480	0.000
Perceived Behavioural Control (PBC)	pbc1	0.702	0.142	4.942	0.000
	pbc2	0.706	0.096	7.320	0.0000
	pbc3	0.750	0.112	6.674	0.000
	pbc4	0.740	0.112	6.634	0.000
Subjective Norm (SN)	sn1	0.876	0.032	27.719	0.000
	sn2	0.811	0.06	13.524	0.000
	sn3	0.839	0.038	22.157	0.000
	sn4	0.736	0.07	10.553	0.000
Personal Moral Norm (PMN)	pmn1	0.916	0.02	45.902	0.000
	pmn2	0.858	0.037	23.074	0.000
	pmn3	0.944	0.01	97.276	0.000
Environmental Concern (EC)	ec1	0.823	0.076	10.801	0.000
	ec2	0.948	0.037	25.762	0.000
	ec3	0.927	0.039	23.926	0.000
	ec4	0.901	0.059	15.314	0.000
Behavioural Intention (BI)	b1	0.727	0.128	5.665	0.000
	b2	0.888	0.061	14.615	0.000
	b3	0.820	0.086	9.550	0.000

**Table 3:** Reliability, Validity, and R^2^ of Constructs.

Construct	Cronbach’s Alpha	Composite Reliability (rho_a)	Composite Reliability (rho_c)	AVE	R^2^	R^2^ Adjusted	f^2^
Attitude (ATT)	0.791	0.841	0.871	0.693	0.072	0.062	0.08 (small–medium)
Behavioural Intention (BI)	0.747	0.775	0.854	0.663	0.166	0.158	0.06–0.08 (small–medium)
Environmental Concerns (EC)	0.922	0.943	0.945	0.812	0.079	0.069	0.08–0.10 (medium)
Perceived Behavioural Control (PBC)	0.707	0.710	0.816	0.525	0.173	0.165	0.15–0.20 (medium)
Personal Moral Norm (PMN)	0.893	0.950	0.932	0.822	0.220	0.213	0.20–0.25 (medium–high)
Subjective Norms (SN)	0.833	0.847	0.889	0.668			

**Table 4:** Structural models: direct and indirect effect estimates on behavioural intentions.

Research Hypothesis	Path	Path Coefficients	Standard Error	*t*-Statistics	*p*-Value
*Direct effects*					
H1	ATT -> BI	0.037	0.141	0.263	0.793
H2a	SN -> BI	0.181	0.127	1.427	0.154
H3	PBC -> BI	0.048	0.101	0.475	0.635
H4a	PMN -> BI	0.234 **	0.116	2.027	0.043
H5	EC -> BI	0.041	0.114	0.359	0.720
*Indirect effects*					
H2b	SN -> PMN -> BI	0.469 ***	0.082	5.710	0.000
H2c	SN -> PBC -> BI	0.416 ***	0.080	5.199	0.000
H4b	PMN -> ATT -> BI	0.268 **	0.109	2.456	0.014
H4c	PMN -> EC -> BI	0.281 ***	0.105	2.676	0.007

** = *p* < 0.05, *** = *p* < 0.01.

## Data Availability

The original contributions presented in this study are included in the article. Further inquiries can be directed to the corresponding author.

## References

[1] FAO (2009). How to Feed the World in 2050.

[2] Echegaray N., Hassoun A., Jagtap S., Tetteh-Caesar M., Kumar M., Tomasevic I., Gulden G., Lorenzo J.M. (2022). Meat 4.0: Principles and applications of industry 4.0 technologies in the meat industry. Appl. Sci..

[3] Barbut S. (2020). Meat industry 4.0: A distant future?. Anim. Front..

[4] Aiking H., de Boer J. (2020). The next protein transition. Trends Food Sci. Technol..

[5] Machovina B., Feeley K.J., Ripple W.J. (2015). Biodiversity Conservation: The Key Is Reducing Meat Consumption. Sci. Total Environ..

[6] Hallström E., Röös E., Börjesson P. (2014). Sustainable meat consumptiona quantitative analysis of nutritional intake, greenhouse gas emissions and land use from a Swedish perspective. Food Policy.

[7] Yadavalli A., Jones K. (2014). Does media influence consumer demand? The case of lean finely textured beef in the United States. Food Policy.

[8] Apostolidis C., McLeay F. (2016). Should we stop meating like this? Reducing meat consumption through substitution. Food Policy.

[9] IARC Working Group (2015). Red Meat and Processed Meat: IARC Working Group on the Evaluation of Carcinogenic Risks to Humans.

[10] Aschemann-Witzel J., Gantriis R.F., Fraga P., Perez-Cueto F.J. (2021). Plant-based food and protein trend from a business perspective: Markets, consumers, and the challenges and opportunities in the future. Crit. Rev. Food Sci. Nutr..

[11] Van Huis A. (2013). Potential of insects as food and feed in assuring food security. Annu. Rev. Entomol..

[12] Mancini S., Moruzzo R., Riccioli F., Paci G. (2019). European consumers’ readiness to adopt insects as food. A review. Food Res. Int..

[13] Mishyna M., Fischer A.R., Steenbekkers B.L., Janssen A.M., Bos-Brouwers H.E. (2023). Consumption and production of edible insects in an urban circularity context: Opinions and intentions of urban residents. Sustain. Prod. Consum..

[14] Tan H.S.G., Verbaan Y.T., Stieger M. (2017). How will better products improve the sensory-liking and willingness to buy insect-based foods?. Food Res. Int..

[15] Verbeke W. (2015). Profiling consumers who are ready to adopt insects as a meat substitute in a Western society. Food Qual. Prefer..

[16] Post M.J. (2012). Cultured meat from stem cells: Challenges and prospects. Meat Sci..

[17] Mancini M.C., Antonioli F. (2019). Exploring consumers’ attitude towards cultured meat in Italy. Meat Sci..

[18] Laestadius L.I., Caldwell M.A. (2015). Is the future of meat palatable? Perceptions of in vitro meat as evidenced by online news comments. Public Health Nutr..

[19] Verbeke W., Sans P., Van Loo E.J. (2015). Challenges and prospects for consumer acceptance of cultured meat. J. Integr. Agric..

[20] Wilks M., Phillips C.J. (2017). Attitudes to in vitro meat: A survey of potential consumers in the United States. PLoS ONE.

[21] Heijnk V., Espey A., Schuenemann F. (2023). A comparison of influencing factors on attitudes towards plant-based, insect-based and cultured meat alternatives in Germany. Food Qual. Prefer..

[22] Willett W., Rockström J., Loken B., Springmann M., Lang T., Vermeulen S., Murray C.J. (2019). Food in the Anthropocene: The EAT–Lancet Commission on healthy diets from sustainable food systems. Lancet.

[23] Hallström E., Carlsson-Kanyama A., Börjesson P. (2015). The environmental impact of dietary change: A systematic review. J. Clean. Prod..

[24] Majcher S. (2025). Consumers’ Perspective of Plant-Based Meat Alternatives—A Systematic Literature Review and Future Research Agenda. Int. J. Consum. Stud..

[25] van derWeele C., Feindt P., van der Goot A.J., van Mierlo B., van Boekel M. (2019). Meat alternatives: An integrative comparison. Trends Food Sci. Technol..

[26] Nolden A.A., Forde C.G. (2023). The nutritional quality of plant-based foods. Sustainability.

[27] Raubenheimer D., Rothman J.M. (2013). Nutritional ecology of entomophagy in humans and other primates. Annu. Rev. Entomol..

[28] Ajzen I. (1991). The theory of planned behavior. Organ. Behav. Hum. Decis. Process..

[29] DeSoucey M. (2010). Gastronationalism: Food traditions and authenticity politics in the European Union. Am. Sociol. Rev..

[30] Bessière J. (1998). Local development and heritage: Traditional food and cuisine as tourist attractions in rural areas. Sociol. Rural..

[31] Steg L., Dreijerink L., Abrahamse W. (2005). Factors influencing the acceptability of energy policies: A test of VBN theory. J. Environ. Psychol..

[32] Stern P.C., Dietz T., Abel T., Guagnano G.A., Kalof L. (1999). A value-belief-norm theory of support for social movements: The case of environmentalism. Hum. Ecol. Rev..

[33] Hosta M., Zabkar V. (2021). Antecedents of environmentally and socially responsible sustainable consumer behavior. J. Bus. Ethics.

[34] Thøgersen J. (2006). Norms for environmentally responsible behaviour: An extended taxonomy. J. Environ. Psychol..

[35] Gautam V., Bhalla S. (2023). Why residents exhibit environmentally responsible behavior?. J. Clean. Prod..

[36] Ajzen I. (2002). Perceived behavioral control, self—Efficacy, locus of control, and the theory of planned behavior. J. Appl. Soc. Psychol..

[37] Taylor S., Todd P. (1995). Decomposition and crossover effects in the theory of planned behavior: A study of consumer adoption intentions. Int. J. Res. Mark..

[38] Ajzen I. (2020). The theory of planned behavior: Frequently asked questions. Hum. Behav. Emerg. Technol..

[39] Hoek A.C., Luning P.A., Weijzen P., Engels W., Kok F.J., De Graaf C. (2011). Replacement of meat by meat substitutes. A survey on person-and product-related factors in consumer acceptance. Appetite.

[40] Siegrist M., Hartmann C. (2020). Consumer acceptance of novel food technologies. Nat. Food.

[41] Loiselle A., Pitre K., Desroches S., Guillaumie L., Bélanger-Gravel A. (2024). Adults’ beliefs related to reducing red meat consumption: An exploratory study in the province of Quebec, Canada. Appetite.

[42] Bamberg S., Möser G. (2007). Twenty years after Hines, Hungerford, and Tomera: A new meta-analysis of psycho-social determinants of pro-environmental behaviour. J. Environ. Psychol..

[43] Yadav R., Pathak G.S. (2016). Intention to purchase organic food among young consumers: Evidences from a developing nation. Appetite.

[44] Vermeir I., Verbeke W. (2008). Sustainable food consumption among young adults in Belgium: Theory of planned behaviour and the role of confidence and values. Ecol. Econ..

[45] Arvola A., Lähteenmäki L., Dean M., Vassallo M., Winkelmann M., Claupein E., Saba A. (2008). Predicting intentions to purchase organic food: The role of affective and moral attitudes in the theory of planned behaviour. Appetite.

[46] Klöckner C.A. (2013). A comprehensive model of the psychology of environmental behaviour—A meta-analysis. Glob. Environ. Chang..

[47] Onwezen M.C., Bartels J., Antonides G. (2014). The self-regulatory function of anticipated pride and guilt in a sustainable and healthy consumption context. Eur. J. Soc. Psychol..

[48] Siegrist M., Hartmann C. (2019). Impact of sustainability perception on consumption of organic meat and meat substitutes. Appetite.

[49] De Backer C.J., Hudders L. (2015). Meat morals: Relationship between meat consumption consumer attitudes towards human and animal welfare and moral behavior. Meat Sci..

[50] Dowd K., Burke K.J. (2013). The influence of ethical values and food choice motivations on intentions to purchase sustainably sourced foods. Appetite.

[51] Rana S.S., Solaiman M. (2023). Moral identity, consumption values and green purchase behaviour. J. Islam. Mark..

[52] Hoek A.C., Pearson D., James S.W., Lawrence M.A., Friel S. (2017). Shrinking the food-print: A qualitative study into consumer perceptions, experiences and attitudes towards healthy and environmentally friendly food behaviours. Appetite.

[53] Hoek A.C., Luning P.A., Stafleu A., De Graaf C. (2004). Food-related lifestyle and health attitudes of Dutch vegetarians, non-vegetarian consumers of meat substitutes, and meat consumers. Appetite.

[54] Sanchez-Sabate R., Sabaté J. (2019). Consumer attitudes towards environmental concerns of meat consumption: A systematic review. Int. J. Environ. Res. Public Health.

[55] Berndsen M., Van der Pligt J. (2004). Ambivalence towards meat. Appetite.

[56] Onwezen M.C., Bouwman E.P., Reinders M.J., Dagevos H. (2021). A systematic review on consumer acceptance of alternative proteins: Pulses, algae, insects, plant-based meat alternatives, and cultured meat. Appetite.

[57] Graça J., Oliveira A., Calheiros M.M. (2015). Meat, beyond the plate: Data-driven hypotheses for understanding consumer willingness to adopt a more plant-based diet. Appetite.

[58] Khine M.S. (2013). Application of Structural Equation Modeling in Educational Research and Practice.

[59] Goldberger A.S., Goldberger M., Eatwell J., Milgate M., Newman P. (1991). Structural equation modeling. The New Palgrave Dictionary of Economics and the Law.

[60] Hair J.F., Ringle C.M., Sarstedt M. (2011). PLS-SEM: Indeed a Silver Bullet. J. Mark. Theory Pract..

[61] Hair J., Page M. (2015). The Essentials of Business Research Methods.

[62] Hair J.F., Hult G.T.M., Ringle C.M., Sarstedt M. (2021). Partial least Squares Structural Equation Modeling (PLS-SEM): Advanced Issues and Applications.

[63] Darlington R.B., Hayes A.F. (2016). Regression Analysis and Linear Models: Concepts, Applications, and Implementation.

[64] Fan Y., Chen J., Shirkey G., John R., Wu S.R., Park H., Shao C. (2016). Applications of structural equation modeling (SEM) in ecological studies: An updated review. Ecol. Process..

[65] Schumacker R.E., Lomax R.G. (2012). A Beginner’s Guide to Structural Equation Modeling.

[66] Diamantopoulos A., Winklhofer H.M. (2001). Index construction with formative indicators: An alternative to scale development. J. Mark. Res..

[67] Rossiter J.R. (2002). The C-OAR-SE procedure for scale development in marketing. Int. J. Res. Mark..

[68] Henseler J., Hubona G., Ray P.A. (2016). Using PLS path modeling in new technology research: Updated guidelines. Ind. Manag. Data Syst..

[69] Jarvis C.B., MacKenzie S.B., Podsakoff P.M. (2003). A critical review of construct indicators and measurement model misspecification in marketing and consumer research. J. Consum. Res..

[70] Henseler J., Ringle C.M., Sarstedt M. (2016). Testing measurement invariance of composites using partial least squares. Int. Mark. Rev..

[71] van Zyl L.E., ten Klooster P.M. (2022). Exploratory Structural Equation Modeling: Practical Guidelines and Tutorial with a Convenient Online Tool for Mplus. Front. Psychiatry.

[72] Nunnally J.C., Bernstein I.H. (1994). Psychometric Theory.

[73] Peterson R.A., Kim Y. (2013). On the relationship between coefficient alpha and composite reliability. J. Appl. Psychol..

[74] Bagozzi R.P., Yi Y. (1988). On the evaluation of structural equation models. JAMS.

[75] Fornell C., Larcker D.F. (1981). Evaluating structural equation models with unobservable variables and measurement error. J. Mark. Res..

[76] Groves R.M., Fowler F.J., Couper M.P., Lepkowski J.M., Singer E., Tourangeau R. (2011). Survey Methodology.

[77] Green S.B. (1991). How many subjects does it take to do a regression analysis?. Multivar. Behav. Res..

[78] Cohen J. (1988). Statistical Power Analysis for the Behavioral Sciences.

[79] Davis F.D. (1989). Perceived usefulness, perceived ease of use, and user acceptance of information technology. MIS Q..

[80] de Castro Carvalho G., Carneiro J.D.D.S., Rocha R.A.R., Pereira E. (2025). Plant-based trends: Consumer’s perception and factors that influence the purchase of plant-based products. Br. Food J..

[81] Ru X., Wang S., Yan S. (2018). Exploring the effects of normative factors and perceived behavioral control on individual’s energy-saving intention: An empirical study in eastern China. Resour. Conserv. Recycl..

[82] De Leeuw A., Valois P., Ajzen I., Schmidt P. (2015). Using the theory of planned behavior to identify key beliefs underlying pro-environmental behavior in high-school students. J. Environ. Psychol..

[83] Kim S.H., Seock Y.K. (2019). The roles of values and social norm on personal norms and pro-environmentally friendly apparel product purchasing behavior: The mediating role of personal norms. J. Retail. Consum. Serv..

[84] Kácha O., van der Linden S. (2021). The moderating role of moral norms and personal cost in compliance with pro-environmental social norms. Curr. Res. Ecol. Soc. Psychol..

[85] Fantechi T., Marinelli N., Casini L., Contini C. (2025). Exploring alternative proteins: Psychological drivers behind consumer engagement. Br. Food J..

[86] Cordano M., Welcomer S.A., Scherer R.F., Pradenas L., Parada V. (2003). A cross-cultural assessment of three theories of pro-environmental behavior. Environ. Behav..

[87] Cavite H.J.M. (2025). Millennial consumers’ intention to purchase organic food: Do environmental concerns matter?. Bus. Strategy Environ..

[88] Cachero-Martínez S. (2020). Consumer Behaviour Towards Organic Products: The Moderating Role of Environmental Concern. J. Risk Financ. Manag..

[89] De Canio F., Martinelli E., Endrighi E. (2021). Enhancing consumers’ pro-Environmental Purchase Intentions: The Moderating Role of Environmental Concern. Int. J. Retail. Distrib. Manag..

[90] Chen S.C., Hung C.W. (2016). Elucidating the factors influencing the acceptance of green products: An extension of theory of planned behavior. Technol. Forecast. Soc. Chang..

[91] Fishbein M., Ajzen I. (2020). Predicting and Changing Behavior: The Reasoned Action Approach.

[92] Onwezen M.C., Antonides G., Bartels J. (2013). The Norm Activation Model: An exploration of the functions of anticipated pride and guilt in pro-environmental behaviour. J. Econ. Psychol..

[93] White K., Habib R., Hardisty D.J. (2019). How to shift consumer behaviors to be more sustainable: A literature review and guiding framework. J. Mark..

[94] Rivis A., Sheeran P., Armitage C.J. (2009). Expanding the affective and normative components of the theory of planned behavior: A meta--analysis of anticipated affect and moral norms. J. Appl. Soc. Psychol..

[95] Siddiqui S.A., Alvi T., Sameen A., Khan S., Blinov A.V., Nagdalian A.A., Mehdizadeh M., Adli D.N., Onwezen M. (2022). Consumer acceptance of alternative proteins: A systematic review of current alternative protein sources and interventions adapted to increase their acceptability. Sustainability.

[96] Lentz G., Connelly S., Mirosa M., Jowett T. (2018). Gauging attitudes and behaviours: Meat consumption and potential reduction. Appetite.

[97] Kaiser F.G. (2006). A moral extension of the theory of planned behavior: Norms and anticipated feelings of regret in conservationism. Personal. Individ. Differ..

[98] Kaiser F.G., Hübner G., Bogner F.X. (2005). Contrasting the Theory of Planned Behavior with the value-belief-norm model in explaining conservation behavior. J. Appl. Soc. Psychol..

[99] Carfora V., Caso D., Conner M. (2017). Randomised controlled trial of a text messaging intervention for reducing processed meat consumption: The mediating roles of anticipated regret and intention. Appetite.

[100] Hartmann C., Siegrist M. (2017). Consumer perception and behaviour regarding sustainable protein consumption: A systematic review. Trends Food Sci. Technol..

[101] Vermeir I., Verbeke W. (2006). Sustainable food consumption: Exploring the consumer “attitude–behavioral intention” gap. J. Agric. Environ. Ethics.

[102] Piazza J., Ruby M.B., Loughnan S., Luong M., Kulik J., Watkins H.M., Seigerman M. (2015). Rationalizing meat consumption. Appetite.

[103] Michel F., Hartmann C., Siegrist M. (2021). Consumers’ associations, perceptions and acceptance of meat and plant-based meat alternatives. Food Qual. Prefer..

[104] Onwezen M.C., Verain M.C., Dagevos H. (2022). Social norms support the protein transition: The relevance of social norms to explain increased acceptance of alternative protein burgers over 5 years. Foods.

[105] Menozzi D., Sogari G., Mora C., Gariglio M., Gasco L., Schiavone A. (2021). Insects as feed for farmed poultry: Are Italian consumers ready to embrace this innovation?. Insects.

[106] Nguyen P., Trieu H.D.X., Le T.B., Le T.D., Tran K.T. (2024). The role of culture, religious belief; subjective norm on the environmental factors and life satisfaction. Int. J. Econ. Policy Emerg. Econ..

[107] Vitell S.J. (2015). A case for consumer social responsibility (CnSR): Including a selected review of consumer ethics/social responsibility research. J. Bus. Ethics.

[108] Shaw D., Shiu E. (2013). The contribution of ethical obligation and selfidentity to the theory of planned behaviour: An exploration of ethical consumers-A reflective comment. Soc. Bus..

[109] Fesenfeld L.P., Maier M., Brazzola N., Stolz N., Sun Y., Kachi A. (2023). How information, social norms, and experience with novel meat substitutes can create positive political feedback and demand-side policy change. Food Policy.

[110] Barbarossa C., De Pelsmacker P. (2016). Positive and negative antecedents of purchasing eco-friendly products: A comparison between green and non-green consumers. J. Bus. Ethics.

[111] Human S.J., Capraro V. (2020). The effect of nudging personal and injunctive norms on the trade-off between objective equality and efficiency. arXiv.

[112] Helferich M., Thøgersen J., Bergquist M. (2023). Direct and mediated impacts of social norms on pro-environmental behavior. Glob. Environ. Chang..

[113] Auger P., Devinney T.M. (2007). Do what consumers say matter? A study of socially responsible consumer behaviour. J. Bus. Ethics.

[114] Fisher R.J. (1993). Social desirability bias and the validity of indirect questioning. J. Consum. Res..

[115] Carrington M.J., Neville B.A., Whitwell G.J. (2014). Lost in translation: Exploring the ethical consumer intention–behaviour gap. J. Bus. Res..

[116] Kollmuss A., Agyeman J. (2002). Mind the gap: Why do people act environmentally and what are the barriers to pro-environmental behaviour?. Environ. Educ. Res..

[117] Carrington M.J., Neville B.A., Whitwell G.J. (2010). Why ethical consumers don’t walk their talk: Towards a framework for understanding the gap between the ethical purchase intentions and actual buying behaviour. J. Bus. Ethics.

[118] Morwitz V.G., Schmittlein D. (1992). Using segmentation to improve sales forecasts based on purchase intent: Which “intenders” actually buy?. J. Mark. Res..

